# Graphitic Carbon Nitride as an Amplification Platform on an Electrochemical Paper-Based Device for the Detection of Norovirus-Specific DNA

**DOI:** 10.3390/s20072070

**Published:** 2020-04-07

**Authors:** Aditya Rana, Manjari Killa, Neelam Yadav, Annu Mishra, Ashish Mathur, Arun Kumar, Manika Khanuja, Jagriti Narang, Roberto Pilloton

**Affiliations:** 1 Amity Institute of Nanotechnology, Amity University, Noida 201313, India; adityarana2116@gmail.com (A.R.); manjari.k11@gmail.com (M.K.); annum407@gmail.com (A.M.); amathur@amity.edu (A.M.); 2 Centre for Biotechnology Maharshi Dayanand University, Haryana 124001, India; neelamindia12@gmail.com; 3 Department of Biotechnology, Deenbandhu Chhotu Ram University of Science and Technology, Haryana 131039, India; 4 Centre for Nanoscience and Nanotechnology, Jamia Millia Islamia University, New Delhi 110025, India; arun.mscphysics@gmail.com; 5 Department of Biotechnology, School of Chemical and Life Sciences, Jamia Hamdard, Hamdard Nagar, New Delhi 110062, India; jags_biotech@yahoo.co.in; 6 National Research Council (CNR), Institute of Crystallography (IC), Via Salaria Km 29.3, Rome I-00015, Italy

**Keywords:** paper-based analytical device, oxidized graphitic carbon nitride, genosensor, methylene blue, norovirus

## Abstract

Norovirus is one of the leading causes of gastroenteritis, acute vomiting, intense diarrhoea, acute pain in the stomach, high fever, headaches, and body pain. Conventional methods of detection gave us very promising results but had disadvantages such as low sensitivity, cost ineffectiveness, reduced specificity and selectivity, etc. Therefore, biosensors can be a viable alternative device which can overcome all setbacks associated with the conventional method. An electrochemical sensor based on oxidized graphitic carbon nitride (Ox-g-C_3_N_4_) modified electrochemical paper-based analytical device (ePAD) was fabricated for the detection of norovirus DNA. The synthesized Ox-g-C_3_N_4_ nanosheets were characterized by field emission scanning electron microscopy (FESEM), X-ray Diffraction (XRD), UV-Vis spectroscopy and X-Ray Photoelectron Spectroscopy. The capture probe DNA (PDNA) modified electrodes were characterized by cyclic voltammetry (CV) and differential pulse voltammetry (DPV). These two characterization techniques were also employed to find the optimal scan rate, response time and temperature of the fabricated sensor. The fabricated biosensor showed a limit of detection (LOD) of 100 fM. Furthermore, the specificity of the reported biosensor was affirmed by testing the response of capture probe DNA with oxidized graphitic carbon nitride (PDNA/Ox-g-C_3_N_4_) modified ePAD on the introduction of a non-complimentary DNA. The fabricated ePAD sensor is easy to fabricate, cost effective and specific, and requires a minimum analysis time of 5 s.

## 1. Introduction

Human norovirus (NoV) is one of the leading causes of gastroenteritis which is also known as infectious diarrhea [1]. It affects all age groups, from children to elderly people, across the whole world [2]. In the United States alone, norovirus affects approximately 56,000 to 71,000 people who, subsequently, need to be hospitalized and around 570–800 of these people die annually [3,4,5]. Food virology research towards the detection of human norovirus has increased manifold after recognizing norovirus as a prominent contributor towards foodborne epidemics [6,7,8]. Conventional methods such as electron microscopy (EM) [9], reverse transcriptase PCR (RT-PCR) assays [10], and enzyme immunoassays (EIAs) [11] were used for detection of the norovirus. These conventional methods gave us very promising results but had their disadvantages such as low sensitivity, cost ineffectiveness, need for experts or professionals to operate the systems, reduced specificity and selectivity [3,12,13]. Hence, biosensors can overcome the shortcomings of the previously used methods of detection such as specificity, sensitivity, and portability [14]. Among them, DNA-based electrochemical biosensors are receiving a lot of attention due to the high specificity of the DNA. Integration of nanomaterials further amplify the response signal which enhances the sensitivity and selectivity of the biosensor [15,16,17]. Biosensors integrated with nanomaterials have shown better stability as well as chemical and electrical properties [18]. Here, graphitic carbon nitride (g-C_3_N_4_) particles were chosen for the interface of the paper electrodes. g-C_3_N_4_ is a semiconducting material which is analogous to graphene with stacked two-dimensional structures [19,20]. The semiconducting 2-D g-C_3_N_4_ has exhibited several advantages including a large surface area, unique physical properties [21], better electrochemical reactions, cost effectiveness, a good source of nitrogen, and excellent chemical stability [22,23]. The presence of nitrogen increases the characteristics of electron donor/acceptor, improves the wettability with the electrolytes, along with the provision of a large additional pseudo capacitance [24,25,26] and exceptional chemical consistency [22,23]. However, direct immobilization of g-C_3_N_4_ has shown some limitations: a fast tendency for recombination with other analytes, lower light consumption efficiency, and thus, restricted the practical applications in both catalysis as well as environmental remediation [20,26]. 

Paper electrodes are advantageous over screen-printed electrodes as they are cheaper and easily manufactured, hence making them suitable for mass production [27,28]. They are unaffected by the conditions of the external environment and do not corrode easily, unlike the screen-printed ones. We report herein the application of electrochemical Paper-based analytical devices (*ePADs*) conjugated with graphitic carbon nitride and DNA probe for the detection of norovirus DNA. DNA Probe employed in the fabrication of the biosensor was the consensus sequence as NoV can be classified as five different distinct geno-groups in order to determine the DNA sequence of all distinct genotypes [29]. The fabricated biosensor showed a low response time, high specificity and selectivity with a low detection limit.

## 2. Experimental Section

### 2.1. Apparatus

The optimization and determination of the analytical performance of the fabricated electrode and sensor were performed using cyclic voltammetry (CV) and differential pulse voltammetry (DPV) on an Autolab-PGSTAT-10. Nova 1.8 software was used for the analysis of all the results. XPS and XRD were done on the respective equipments.

### 2.2. Reagents

The synthesis of Ox-g-C_3_N_4_ was carried out by using Melamine (10g) in H_2_SO_4_ (98%) and HNO_3_ (69%), deionized water, H_2_O_2_ (33%), methylene blue (MB), and potassium chloride (KCl). The 18-mer oligonucleotides were purchased from GCC Biotech, India. The base sequences were as follows:

Capture probe—5’ TATGTTGACCCTGATAC-3′ 

Target probe—3’ GTATCAGGGTCAACATA-5’

The solutions of different concentrations of oligonucleotides were made by using TE buffer solution (10 mM Tris-HCl, 1 mM EDTA, pH 7.2) and then they were kept in a refrigerator at 4 °C to prevent degradation [14].

### 2.3. Synthesis of g-C_3_N_4_ (GCN)

First, to prepare g-C_3_N_4_, melamine was heated thermally in a muffle furnace at a temperature of 550 °C for a duration of 3 h at 5 °C/min to obtain a yellowish powder [30]. Afterwards, the obtained powder (2 g) was added into a 40 mL mixture solution which consisted of concentrated H_2_SO_4_ (98%) and HNO_3_ (69%) in a ratio of 1:1. This solution mixture was further sonicated at a temperature of 40 °C for 2 hours. To the obtained solution, 3 mL of H_2_O_2_ (33%) was added drop-wise and then further sonicated for an additional 3 hours to exfoliate bulk g-C_3_N_4_ and a whitish-yellow product was obtained. An amount of 150 mL of deionized water was then added to the resulting mixture. This created a diluted mixture of the oxidized g-C_3_N_4_ (Ox-g-C_3_N_4_), which was further centrifuged at approximately 10,000 rpm. The resulting mixture was then washed with acetone or alternatively, deionized water (DI), after which it was dried at a temperature of 70 °C for 10 hours. At the end, we obtained exfoliated yellow colored nanosheets of Ox-g-C_3_N_4_.

### 2.4. Fabrication of ePADs Grafted with Ox-g-C_3_N_4_ Nanosheets

For the fabrication of the electrochemical paper-based analytical devices (ePADs), cellulose sheets were used as the substrate. A standard screen-printing technique was used to print the electrodes onto the cellulose paper. Conductive carbon ink was used for grafting electrodes onto the surface of the paper. The printed electrode ink was then dried at room temperature for a couple of minutes. The design of the electrodes is shown below in the schematic diagram 1. To make the electrodes an electrochemical cell, hydrophobic barriers were made on the electrodes, which also prevented the flow of the electrolyte outside areas of the working region. The well was developed using wax coating and then the paper electrodes were kept at 60 °C for 10 min. The heating ensured that the wax would flow through the thickness of the paper electrodes and behave as a hydrophobic barrier. It was confirmed that each of the electrodes had an identical conductivity before using them for any electrochemical analysis with the help of a multi meter. Afterward, prepared dispersed solution of Ox-g-C_3_N_4_ nanoparticles (2 μL; 1 mg/μL) was drop casted carefully onto the surface of the electrode. Then the electrodes were kept at room temperature for air-drying and to form a smooth coating of the nanoparticles.

### 2.5. Fabrication of PDNA/Ox-g-C_3_N_4_/ePAD

The capture probe DNA (PDNA), which is a single-stranded DNA, was first immobilized by physio-sorption. The capture probe DNA (5’ GATGAGTATTGATGCCGA 3’) was prepared with TE buffer (pH 7.2). Then, the PDNA solution was introduced onto the surface of the working electrode of the fabricated sensor. The probe was immobilized by drop-casting 5 µL of the PDNA over the circular working region of ePAD and keeping the platforms at 3 °C for 30 min. The fabricated PDNA/Ox-g-C_3_N_4_/ePAD was checked for electrochemical performance by CV in the potential range of −1.0 to +1.0 V in a solution containing 0.1 mM methylene blue in KCl. Various other parameters such as scan rate, response time and temperature were optimized (See supplementary information). The optimization was done to obtain maximum signal of Ox-g-C_3_N_4_ modified ePAD. The PDNA/Ox-g-C_3_N_4_/ePAD were then left for 2–3 h at room temperature.

### 2.6. Hybridization of Target DNA 

The fabricated electrodes were tested for different concentrations of the target DNA (TDNA) (3’ TCGGCATCAATACTCATC 5’) ranging from 100 fM to 100 µM. The volume of target DNA that was drop casted on each electrode was 5 µL (the same as probe) immediately before performing electrochemical studies. Moreover, the specificity of the fabricated sensor was tested by introducing a non-complimentary DNA and checking the electrochemical response of the developed sensor.

## 3. Results and Discussions

### 3.1. Ox-g-C_3_N_4_ Nanosheets Characterization

**Figure 1:**
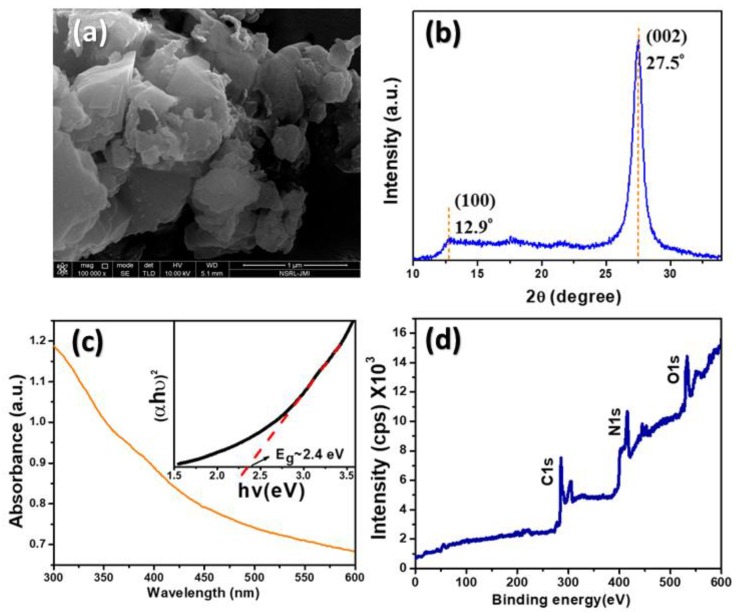
(**a**) FESEM image of Ox-g-C_3_N_4_ (**b**) XRD peaks of Ox-g-C_3_N_4_ (**c**) UV-visible absorbance spectra (inset: Tauc’s plot) of Ox-g-C_3_N_4_ and (**d**) XPS survey scan of Ox-g-C_3_N_4._

The surface morphology of the as-prepared sample was analyzed using field emission scanning electron microscopy (FESEM), as shown in Figure 1a. The morphology was found to be irregular nanosheets similar to a graphite-like structure. The XRD pattern of Ox-g-C_3_N_4_ is shown in Figure 1b. The observed characteristic peaks of Ox-g-C_3_N_4_ that appeared at 12.9° and 27.5° correspond to the (100) and (002) plane, with an observed interlayer spacing of 0.685 nm and 0.325 nm, respectively [28,31]. This is in good agreement with the literature values of JCPDS No. (87–1526). The optical properties of samples were carried out by the UV-Vis absorption spectra and the result was shown in Figure 1c. The optical bandgap (E_g_) was successfully calculated using tauc’s plot, which is found to be 2.4 eV, as shown in the inset. XPS is a very powerful technique to study the elemental and oxidation states of a sample, as shown in Figure 1d. In the XPS survey scan, peaks were observed at 286.93 eV, 405.7 eV, 530.1 eV; these corresponded to carbon (C1s), nitrogen (N1s), and oxygen (O1s) elements respectively.

### 3.2. Electrochemical Response of Ox-g-C_3_N_4_ Modified Electrodes

**Figure 2:**
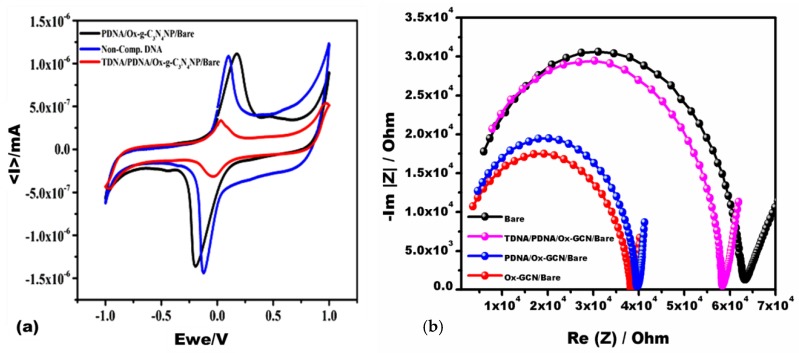
(**a**) CV at various stages including Bare, Ox-g-C_3_N_4_ modified, PDNA/Ox-g-C_3_N_4_/ePAD, TDNA/Ox-g-C_3_N_4_/ePAD conducted in 0.1 mM KCl containing 0.1 mM MB (pH 7.2) (Potential 1 mV) at the scan rate of 100 mV/s and (**b**) Nyquist plot at various stages of ePAD including Bare, Ox-g-C_3_N_4_ modified, PDNA/Ox-g-C_3_N_4_/ePAD, TDNA/Ox-g-C_3_N_4_/ePAD conducted in '0.1 mM KCl containing 0.1 mM MB (pH 7.2) at the frequency range of 100 Hz–10^3^ KHz.

Cyclic voltammetry was conducted to electrochemically study the different stages of the electrode in 0.1 mM KCl containing 1 mM methylene blue. The difference in the peak currents of MB was kept as the basis of the electrochemical study. As can be seen from Figure 2a, a bare ePAD shows a well defined cathodic and anodic peak. This peak is less when compared to Ox-g-C_3_N_4_ modified ePAD due to the conductive nature of Ox-g-C_3_N_4_. In addition, the redox potential is also shifting towards the slightly higher side, which can also further confirm the immobilization of Ox-g-C_3_N_4_. The increase in the anodic current and slight shifting of potential can be due to the fact that the high surface area of Ox-g-C_3_N_4_ provided more active sites, which produces a high current. However, there is a slight or insignificant change in potential of about 0.02 V, which can be due to its semi-conductive nature. After the immobilization of PDNA, the peak current decreased, which is due to the non-conductive behavior of PDNA. This (PDNA/Ox-g-C_3_N_4_/ePAD) signal peak is greater in comparison to the response of hybridized target DNA (TDNA/Ox- C_3_N_4_/ePAD) because the methylene blue (MB) redox indicator gets intercalated between the double-stranded DNA. This can be attributed to the fact that the target DNA (TDNA) successfully hybridized with the capture probe DNA (PDNA). The EIS study is depicted in Figure 2b, which demonstrates the change in resistance charge transfer (Rct) at various stages of electrode fabrication. The bare electrode shows increased Rct as compared to the nanoparticles-modified electrode. After the immobilization of DNA-probe-modified electrode, there was an increase in Rct, but after hybridization with the target DNA, there was a further increase in Rct. The CV results are in line with the EIS results. 

### 3.3. Analytical Performance of the Developed Biosensor

**Scheme 1:**
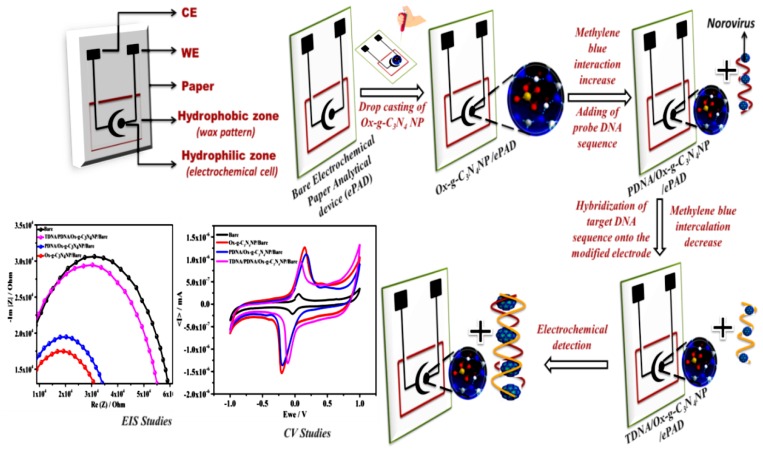
Schematic representation of the fabricated biosensor.

The schematic representation of Ox-g-C_3_N_4_ integrated electrochemical paper-based analytical device (ePAD) fabricated for the detection of norovirus DNA is depicted in Scheme 1.

**Figure 3:**
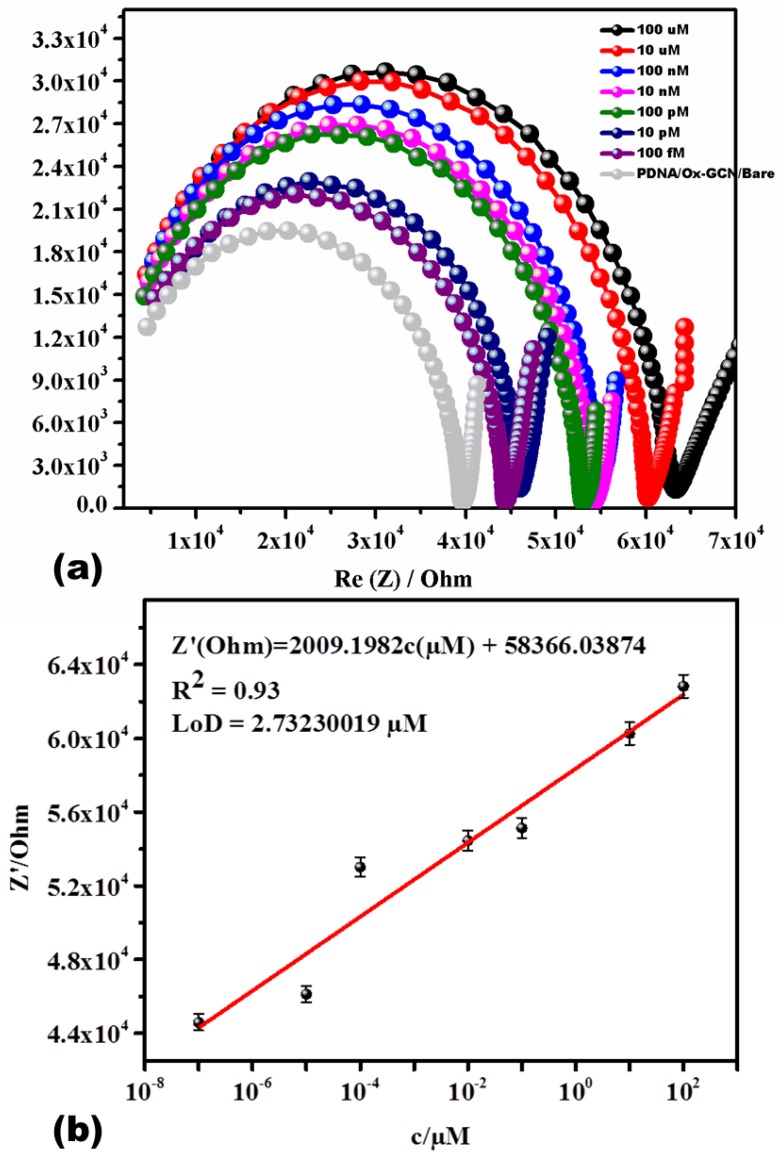
(**a**) Nyquist plot confirmed hybridization of different concentrations of the complimentary target DNA at PDNA/Ox-g-C_3_N_4_ nanosheets/Bare using '0.1 mM KCl containing 0.1 mM MB (pH 7.2). (**b**) The calibration plot of the TDNA/PDNA/Ox-g-C_3_N_4_ nanosheets/Bare electrode as a function of the logarithmic concentration of the Target DNA and change in resistance charge transfer.

For the hybridization study, different concentrations of complimentary target DNA ranging from 100 fM to 100 μM were chosen and introduced on PDNA/Ox-g-C_3_N_4_-coated paper electrodes. As depicted in Figure 3, the current signal of MB decreases with the increase in the concentration of target DNA (TDNA). Methylene blue is a cationic dye containing two amino group which contributed positive charge to the dye and positively charged dye has easily shown electrostatic interaction with the free guanine bases of ssDNA. Once the ssDNA is hybridized with the target DNA, there will be less accessibility of free guanine bases as duplex formation prevents the electrostatic interaction and hence, the signal is reduced [15,16]. This can be attributed to the fact that with an increase in concentration of target DNA, less free guanine base pairs were available for showing interactions with the MB and with decreased concentration more free guanine bases were available for interaction with MB, as a smaller amount of capture probe DNA is hybridized. The response signal in the form of current decreased as there was an increase in the target DNA (TDNA) concentration due to intercalation of MB in between the oligonucleotides of the double-stranded DNA (TDNA) [32]. The EIS results show that with an increase in target concentration, there was more Rct depicted in Figure 3a. Figure 3b shows the calibration plot of the TDNA/PDNA/Ox-g-C_3_N_4_/Bare electrode in 0.1 mM KCl containing 1 mM methylene blue. 

### 3.4. Selectivity of the Developed Biosensor

**Figure 4:**
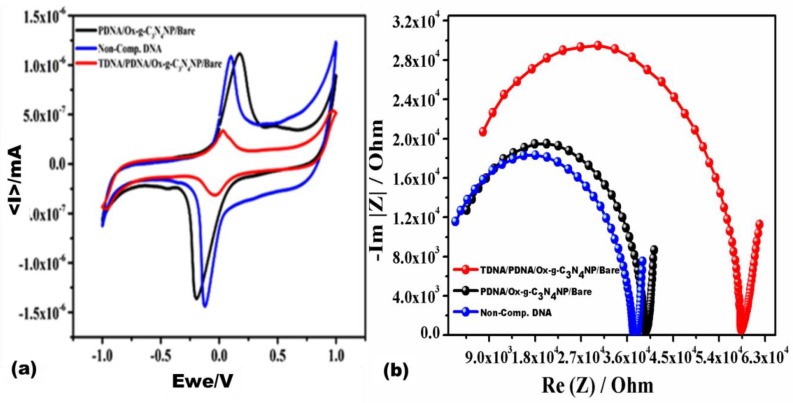
(**a**) CV of (**i**) PDNA/Ox-g-C_3_N_4_/ePAD (**ii**) PDNA/Ox-g-C_3_N_4_/ePAD+ non complementary DNA (iii) PDNA/Ox-g-C_3_N_4_/ePAD + complementary DNA (**b**) Nyquist plots.

The PDNA/Ox-g-C_3_N_4_ nanosheets-coated paper electrodes were also tested for selectivity by comparing the electrochemical response of the electrodes hybridized with double-stranded DNA (TDNA) and electrodes hybridized by non-complimentary single-stranded DNA (10 μM) (PDNA). This was performed by introducing both target DNA and non-complimentary DNA into the working area of the electrodes modified with capture probe DNA (PDNA) and Ox-g-C_3_N_4_ nanosheets. As shown in Figure 4, there is no current decrease in the case of non-complimentary DNA. 

On the other hand, MB shows a differential sensing signal with single-stranded DNA, capture-probe DNA and non-complimentary DNA. Thus, the fabricated biosensor was highly selective to human norovirus-specific DNA. Table 1 shows the comparison between various biosensors integrated with different types of electrodes for the investigation of norovirus.

## 4. Conclusions

It can be concluded that a norovirus electrochemical DNA biosensor was successfully developed by incorporating Ox-g-C_3_N_4_ nanosheets onto paper electrodes. Paper electrodes have the potential to be sensing substrates that are cost effective, portable, as well as disposable. In addition to this, they are extremely useful in limited resource settings. The developed biosensor showed high selectivity and sensitivity towards norovirus DNA. Ox-g-C_3_N_4_ nanosheets are offering biocompatibility and an increased sensing signal but nanoparticles need to be optimized further in order to increase the stability response for future studies. The limit of detection of the fabricated biosensor was found to be 100 fM, which shows that the fabricated biosensor was highly sensitive and specific towards the target norovirus DNA. 

## Figures and Tables

**Table 1:** Comparison of various biosensors integrated with different types of electrodes used for norovirus detection.

Working Electrode	Temperature (°C)	Limit of Detection (LOD)	Linear Range	Response Time (minutes)	References
Screen Printed Gold electrode	-	1.7 copies/mL	0–10^5^ copies/mL	30	[33]
Gold electrode	-	35 copies/mL	10^2^–10^6^ copies/mL	60	[34]
Paper-based electrode	-	4.4 ng/mL–3.3 ng/mL	13 ng/mL–13 µg/mL	-	[35]
Localized surface plasmonic resonance (LSPR)	25	9.9 copies/mL	0.001–100 µg/mL	10	[36]
Gold electrode	-	7.8 copies/mL	-	-	[37]
CM_3_ Sensor chip	25	104 TCID_50_ FCV/ML		15	[38]
LSPR		84 copies/mL	1 pg/mL to 5 ng/mL	-	[39]
V-trench sensor chip	-	0.01 ng/mL	0–100 ng/mL	1	[40]

## Supplementary Materials

The following are available online at https://www.mdpi.com/1424-8220/20/7/2070/s1, Figure S1: (a) Differential pulse voltammetry (DPV) at various response times ranging from 5–30 sec (b) DPV of PDNA/Ox-g-C3N4/ePAD at different temperatures ranging from 15–55 °C (c) Scan rate optimization ranging from 10 to 100 mV/s. (d) Linear plot of current vs. log V. All optimization studies were done using '0.1 mM KCl containing 0.1 mM MB (pH 7.2).
