# Supplementary material for: Graphitic Carbon Nitride as an Amplification Platform on an Electrochemical Paper-Based Device for the Detection of Norovirus-Specific DNA
Source: Sensors (Basel). 2020 Apr 7;20(7):2070. doi: 10.3390/s20072070 (PMC7180435; doi:10.3390/s20072070)
Supplement: Supplementary file 1 [file sensors-20-02070-s001.pdf]

Optimization of Various Parameters

Various parameters of the experiment such as response time, temperature and scan rate were optimized to achieve the best sensing signals. Different response times in the range of 5 to 30 s were taken for the electrochemical analysis. As is evident from Figure S1a, the current signal peak climbed up as the response time increased, but after 25 s, there is a drastic decrease in the current. The PDNA/Ox-g-C_3N_4 coated paper electrodes were further tested at varying temperatures from 15 °C to 55°C. The optimization of temperature is important as it affects the stability of DNA. As is evident from Figure S1b, the current signal peak increases with the increase in temperature until the temperature reaches 45 °C, after which there is a decrease in the peak. From this study, we found that 45°C was the optimal temperature and was thus selected for hybridization. Cyclic voltammetry (CV) of PDNA/Ox-g-C_3N_4 coated paper electrodes was done at different scan rates varying from -1.0 to + 1.0 V/s (from 10 mV/s to 100 mV/s), Figure S1c and was studied for optimization. This was done to achieve optimal performance and stability of the electrode at different scan rates. Figure S1d shows that with increasing scan rate, the current is also increased. The fabricated electrodes were stable which is evident from the fact that the ratio of anodic current peak (I_a) and cathodic current peak (I_c) is almost close to unity. Thus, the fabricated electrodes show stability.

The linear equation in the peak current (I) with log (V) is shown in Figure S1d, which can be expressed in the form of an equation as given below:

$$I = -3.7 \text{ X } 10^{-4}x + 8.1 \text{ X } 10^{-4}x \ (r^2 = 0.99)$$

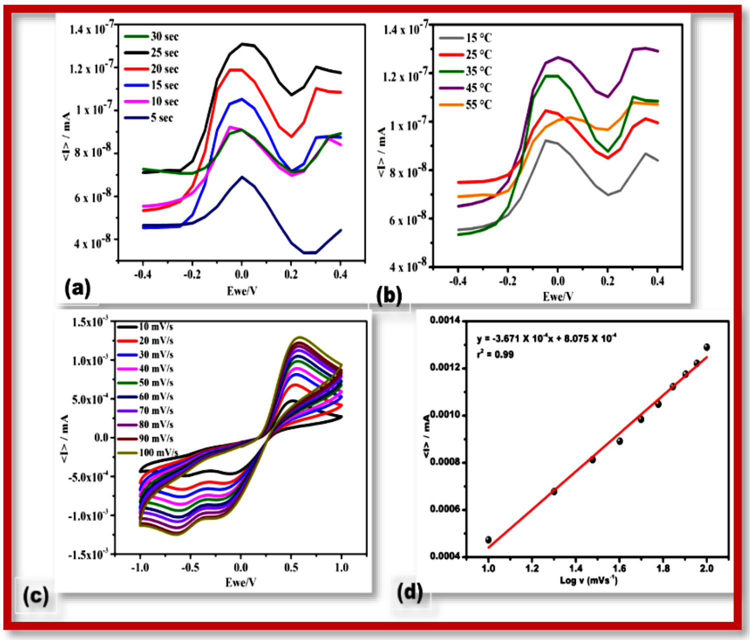

Figure S1. (**a**) Differential pulse voltammetry (DPV) at various response times ranging from 5–30 sec (**b**) DPV of PDNA/Ox-g-C_3N_4/ePAD at different temperatures ranging from 15–55 °C (**c**) Scan rate optimization ranging from 10 to 100 mV/s. (**d**) Linear plot of current vs. log V. All optimization studies were done using 0.1 M MB (pH 7.2) containing 0.1 M KCl.
